# Evaluation of Dietary Intakes and Supplement Use in Paralympic Athletes

**DOI:** 10.3390/nu9111266

**Published:** 2017-11-21

**Authors:** Robyn F. Madden, Jane Shearer, Jill A. Parnell

**Affiliations:** 1 Department of Biochemistry and Molecular Biology, Faculty of Kinesiology, Cumming School of Medicine, University of Calgary, 2500 University Drive NW, Calgary, AB T2N 1N4, Canada; robyn.madden1@ucalgary.ca (R.F.M.); jshearer@ucalgary.ca (J.S.); 2 Department of Health and Physical Education, Mount Royal University, 4825 Mount Royal Gate SW, Calgary, AB T3E 6K6, Canada

**Keywords:** Paralympic athlete, nutrient intakes, dietary supplements, nutritional information

## Abstract

Dietary intakes and supplement use in Paralympic athletes remains largely unexplored, and specialized recommendations are lacking. The aim of this study was to evaluate nutrient intakes and supplement use in high-performance athletes with physical disabilities using three-day food records and a validated dietary supplement use questionnaire. A secondary aim examined gender differences in nutrient and supplement intakes. Male (*n* = 18) and female (*n* = 22) athletes were recruited from nine Paralympic sports through sporting organizations, coaches, and social media. Athletes generally met able-bodied recommendations for macronutrients. Male and female athletes often failed to meet the Recommended Dietary Allowance (RDA) or Adequate Intake (AI) for vitamin D, vitamin E, pantothenic acid, magnesium, and potassium. On average, females did not meet the RDA for iron and calcium, whereas males did not meet the RDA for vitamin A and folate. Commonly consumed supplements were vitamin D, protein powder, sport bars, and sport drinks. Analysis of diet and supplement use within this population shows several micronutrient deficiencies and irregular use of specific supplements. Athlete support and education is required to optimize nutrition in Paralympic athletes.

## 1. Introduction

The Paralympic games are highly competitive, and athletes need to maximize performance—one crucial aspect of which is nutrition. Proper sports nutrition should be incorporated into an athlete’s lifestyle, beginning at the training phase. Specifically, athletes should focus on optimal amounts and timing of macronutrient consumption while ensuring daily micronutrient recommendations are met [1]. Incorporating a proper diet full of nourishing foods may enhance an athlete’s overall health status, which can help to decrease the risk of injury or illness [2]. Ultimately, proper sports nutrition ensures that years of training and preparation do not go to waste.

Sport nutritional needs are non-transferable between athletes and are dependent upon the type, duration, and intensity of the activity performed [3]. The addition of a physical impairment further challenges and complicates nutritional recommendations. For example, wheelchair athletes have reductions in muscle mass and sympathetic nervous system, as compared to able-bodied (AB) athletes, resulting in lower energy requirements [4]. Even with the recent increase in popularity of Paralympic sport, few studies have focused on the sport nutrition needs and behaviors of these athletes [5]. With no general nutritional guidelines, these athletes are forced to default to able-bodied recommendations.

This lack of nutritional guidelines, combined with the fact that athletes with impairments generally have lower energy intakes, may lead to low levels of specific micronutrients; therefore, dietary supplementation (DS) may be necessary [1]. Although there is little information regarding supplement use in athletes with disabilities, a few studies have investigated the types, prevalence, and reasoning of DS among this population. Currently, the literature shows large variations in dietary supplement consumption in athletes with an impairment, depending on the demographic and supplements included [5,6,7]. Reasons for DS have been reported as “exercise recovery”, “provide energy”, “increase strength/power”, “support immune system”, “medical need/deficiency”, and “inadequate diet” [5]. Few studies have reported sources of nutritional information, though dieticians have been reported as a main source [5].

With little research surrounding nutrition in athletes with physical disabilities, the primary objective of this study was to assess macro- and micronutrient intakes and types of dietary supplements being consumed. The secondary objective was to report preferred sources of nutritional information for athletes with impairments. Awareness of dietary concerns, types of dietary supplements, and preferred sources of information can be used to develop educational programs and guide future research.

## 2. Materials and Methods

### 2.1. Participants

Male and female athletes 18 years and older who met the criteria for an athlete with a physical or visual disability, as set out by the International Paralympic Committee (IPC) [8], were recruited from nine Paralympic sports through sporting organizations, coaches, and social media. The athletes were instructed to email the researchers if they were interested in participating after seeing recruitment information, either through social media or email from their coach or sporting organization. Athletes with impaired muscle power, impaired passive range of movement, limb deficiency, leg length difference, short stature, hypertonia, ataxia, athetosis, or vision impairment [8] were eligible to participate in the study. Exclusion criteria included athletes who did not speak English or who were classified as having an intellectual impairment, as the questionnaires were not validated for these populations.

The study was approved by the Mount Royal University Human Research Ethics Board (Ethics ID 2015-35). Athlete written consent was provided prior to completing the Dietary Supplement Questionnaire [9,10] (Supplementary Files: S1 Dietary Supplement Questionnaire) and Three-Day Food Records (Supplementary Files: S2 Three Day Food Records).

### 2.2. Three-Day Food Records

Dietary intakes were collected via Three-Day Food Records—a protocol tested for validity and reliability and previously used in athletes with spinal cord injuries [7]. Athletes were to indicate their age, height, weight, whether or not they were vegetarian, competitive sport, and current training phase. Athletes were instructed to record all food and beverages consumed during three subsequent days of their choosing. Vitamin and mineral supplements were not included in the food records; however, protein powders, sport beverages, and sport bars were included. To ensure accuracy, athletes were encouraged to maintain their normal eating habits during the three days. A video tutorial link (https://www.youtube.com/watch?v=Hpra2GoyWRA&feature=youtu.be) [11] was provided to help estimate portion sizes.

The Three-Day Food Records were analyzed using FoodWorks version 17 (The Nutrition Company, Long Valley, NJ, USA). FoodWorks derives the nutritional information for its food sources from over 40,000 food references, including USDA Standard Reference 27, the Food and Nutrition Database for Dietary Studies, and the Canadian Nutrient File 2010. Additionally, direct data from seven fast food restaurants was used. A complete nutrient list can be found at http://www.nutritionco.com/FWnutrients.htm [12]. When a food was not found in the database, it was manually added using its food label. Averages from each participant’s three days were used for the analysis.

### 2.3. Dietary Supplement Questionnaire

A dietary supplement questionnaire [9,10] was used to assess dietary supplement intakes and sources of supplement information. First, it identified the athlete’s age, gender, sporting event, current training phase, top level of competition, and hours of training per week. Second, it quantified supplement use, reasons for using supplements, and which sources of information athletes use to receive information about supplements. Athletes were also asked what their preferred sources of nutritional information were. Supplement usage was comprised of a list of 25 supplements including vitamins, minerals, fortified and sport beverages, protein powders, sport (carbohydrate-dense or protein-dense) bars and gels, fatty acids, plant extracts, and probiotics. If a supplement was not listed, space was provided to add it. Athletes indicated how often they use each type of supplement, using five options: regularly, specific times, tried, never, and unfamiliar [10]. If an athlete indicated usage of a supplement, they were encouraged to provide brand name and dosage beside it. Lastly, the questionnaire asked each athlete to rank their overall diet as “not very healthy”, “pretty healthy (average)”, or “very healthy”.

### 2.4. Procedures

Participants were mailed or emailed a copy of the consent form, along with the Dietary Supplement Questionnaire and Three-Day Food Records, and a postage paid return envelope.

### 2.5. Statistical Analyses

Athletes were categorized into groups based on gender: males 18–50 years and females 18–50 years. Intakes of macronutrients are presented as absolute values, percent of total calories, and grams per kilogram of body weight. Percent of total calories for carbohydrates, sugar, and protein was determined by multiplying the grams by 4 kcal/g, dividing by the total calories, and multiplying by 100. Calculations that adjust for dietary fiber were not conducted, as there is no clear consensus on the energy value per gram of dietary fiber. Percent of total calories from fats was calculated by multiplying the grams by 9 kcal/g, dividing by the total calories and multiplying by 100. All micronutrient intakes are presented as the percent of the Recommended Dietary Allowance (RDA) or Adequate Intake (AI). Percent of the Tolerable Upper Intake Levels (UL) was included for sodium. Percent RDA, AI, and UL were calculated by taking each athlete’s intake and dividing it by the established RDA, AI, or UL for their age and gender [13] and multiplying by 100. Absolute values for micronutrients are not presented, as the categories span multiple age groups. Responses from the dietary supplement questionnaire were condensed into three categories and quantified as the percent of respondents who consumed the supplement Regularly (“at least 2 times per week”), Occasionally (“Specific Times” or “Tried it”), and Never (“Never” or “Unfamiliar”) [10]. A Shapiro–Wilk test was used to check data for normality. Means and standard deviations were determined for normally distributed data and medians and interquartile ranges for non-normal data. Differences between genders in energy, macronutrient, and micronutrient intakes were determined by an independent *t*-test for normally distributed data or Mann–Whitney U test for data that was not normally distributed. Differences between genders in supplement use were determined by a chi square test and sources of information using a Fisher’s exact test. All analyses were performed using SPSS Statistics version 24 (IBM Corporation, Armonk, NY, USA).

## 3. Results

### 3.1. Participant Characteristics

A total of 42 athletes were sent questionnaires, and 40 athletes responded. Descriptive characteristics of the participants are outlined in Table 1.

### 3.2. Nutrient Intakes

Macronutrient intakes are presented in Table 2. Males had significantly greater total energy intakes than females (male = 2092 kcal/day; female = 1602 kcal/day, *p* = 0.013). There were no differences in carbohydrate and fat intakes based on body weight (BW) between genders. Males had greater protein intakes based on body weight at 1.6 g/kg BW compared to females at 1.4 g/kg BW (*p* = 0.017). Females consumed a significantly greater percentage of their total calories from sugar as compared to males (*p* = 0.049).

Micronutrient intakes are presented in Table 3. Overall, athletes’ intakes met or exceeded the majority of RDAs for vitamins and minerals, with the exception of vitamin D, vitamin E, and magnesium. When analyzed by gender, males did not meet the RDA for folate and vitamin A, while females did not meet the RDA for iron and calcium. Athletes did not meet the AI for pantothenic acid and potassium. Sodium intakes exceeded the upper limit in both genders. Significant differences were noted, as females had lower percent of the recommendations for calcium (*p* = 0.031), selenium (*p* = 0.016), and iron (*p* < 0.001) as compared to males.

When athletes were asked to rank their diet as a whole, 11% of males and 5% of females ranked their diet as “not healthy”, 61% of males and 73% of females ranked their diet as “average”, and 28% of males and 23% of females thought their diet was “very healthy”. There was no difference between genders (*p* = 0.648).

### 3.3. Supplement Use

**Figure 1:**
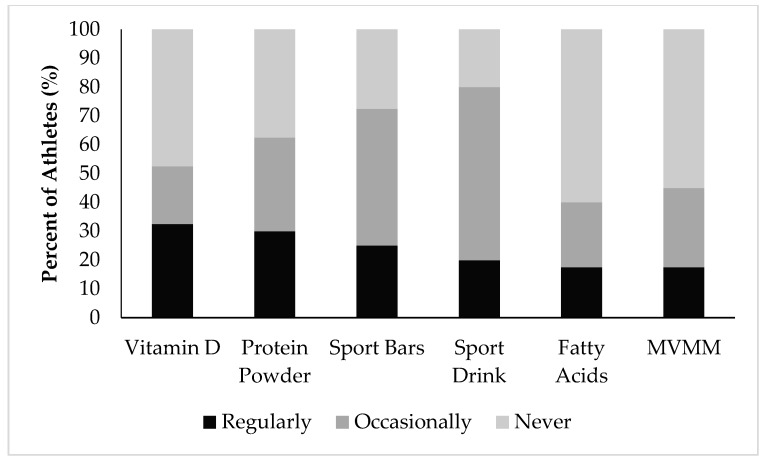
Dietary supplements commonly consumed by Paralympic athletes. Sport bars included carbohydrate and protein based bars designed for athletes. Sport drinks included electrolyte/carbohydrate formulas and electrolyte formulas. MVMM, multivitamin multimineral formulations.

Athlete use of dietary supplements is presented as those who consume the supplement regularly, occasionally, or never over the past three months (Figure 1). The majority of athletes used at least one supplement over the past three months (males = 100%; females = 91%, *p* = 0.492).

Males most regularly used sport bars (38.9%), protein powder (38.9%), and sport drinks (33.3%), whereas females most regularly used vitamin D (40.9%), protein powder (22.7%), and fatty acids (18.2%).

When analyzed according to gender, males were more likely to use branched chain amino acids (BCAAs) (*p* = 0.008). There were no other significant differences between genders in supplement use. Complete results for dietary supplement use are provided in Supplementary Files: S6 Dietary supplements and ergogenic aids.

When asked “What are your reason(s) for taking dietary supplements?” athletes reported their top five reasons as “stay healthy” (50.0%), “increase energy” (42.5%), “medical” (40.0%), “enhance overall athletic performance” (37.5%), and “improve exercise recovery” (37.5%). Males reported their top three reasons as “improve exercise recovery” (50.0%), “increase energy” (44.4%), and “enhance overall athletic performance” (44.4%). Females’ top reasons for taking dietary supplements were “stay healthy” (59.1%) and “medical (your doctor told you to)” (50.0%). There were no significant differences between males and females in reasons for supplementation.

### 3.4. Sources of Information

When asked the question “Where do you get information about dietary supplements?” athletes reported their top three sources as dietician/nutritionist (62.5%), medical physician (doctor) (40%), and athletic trainer (27.5%). Both genders reported dietician/nutritionists as their number one source. Females reported medical physician (doctor) (45.5%) and product labels (18.2%) as their second and third choices. Males reported athletic trainer (50.0%) as their second choice and medical physician (doctor) (33.3%), internet (33.3%), and teammates/friends (33.3%) tied for third. Males were significantly more likely to get information from athletic trainers than females (*p* = 0.006). The least common sources for both genders included pharmacist (2.5%), television (5%), naturopath/chiropractor (7.5%), physio/massage therapist (7.5%), health store (7.5%), and print media (7.5%).

When the question was changed to “Which way do you prefer to receive information about dietary supplements?” athletes preferred “individual nutrition consultation” (80%), “coach or athletic trainer” (45%), and “doctor/chiropractor/physiotherapist, etc.” (45%). Thirty-three percent of males preferred to receive information from “family/friends” as compared to 4.5% of females (*p* = 0.033). Though not significant, “coach or athletic trainer” should be noted, as 61.1% of males reported preferring this source, whereas only 31.8% of females preferred this source for nutritional information (*p* = 0.110).

## 4. Discussion

This research analyzes dietary intakes and supplement use in elite Canadian athletes with physical disabilities, an understudied population, and highlights areas of potential dietary improvements.

### 4.1. Overall Diet Quality and Energy Intakes

Our findings show that elite Canadian athletes with physical disabilities tend to consume a diet that is adequate in most macro- and micronutrients. Female energy intakes were significantly lower when compared to males—a trend that has been cited in previous literature [14]. Total energy intakes, in athletes with impairments have been reported to differ significantly from athlete to athlete [1,7,14], solidifying the need for individualized nutrition plans for each athlete.

Athletes’ energy needs will fluctuate during seasonal training phases, and are often higher than energy intakes in able-bodied endurance athletes [15]. When compared to their AB peers, athletes with physical impairments may have lower total energy requirements. For example, athletes with paralysis have less mobility and active muscle mass than their AB peers, potentially leading to reduced energy expenditure. Consequently, these athletes may need to consume less food to maintain energy balance [1]. In the absence of research indicating a lower need for micronutrients, the assumption is that food choices need to be more nutrient-dense in athletes with impairments in order to meet vitamin and mineral requirements with a lower energy intake.

### 4.2. Macronutrients

The standardization of macronutrient recommendations for athletes with physical disabilities will be complicated since nutritional needs vary from athlete to athlete. Despite the challenges, some guidelines should be developed, similar to those available for AB athletes [3]. In the absence of guidelines for impaired athletes, we have deferred to AB recommendations as a proxy; however, limitations are acknowledged. Carbohydrate recommendations for AB athletes suggest that consumption should be based on body weight and typically will range from 3 to 12 g/kg BW depending on exercise type, volume, etc. [16]. Based on this range, minimum carbohydrate recommendations were met in both genders, as both male and female athletes had mean intakes of 3.5 g/kg BW, which is line with others [1,17]. Depending on impairment, emphasizing higher carbohydrate intakes may be necessary. However, carbohydrates should be monitored since an increase in carbohydrate consumption may lead to weight gain if it results in excessive calories [14]. Fiber intakes did not meet the recommendations in either gender group. Conversely, sugar intakes were 18% and 22% of total calories for males and females, respectively. The values represent total sugars, and include naturally present and added sugars. Currently we are unaware of any recommendations for total sugar intake; however, the World Health Organization makes a conditional recommendation of “free sugars” intake below 5%, although this is not athlete-specific [18]. Regardless, the combination of low fiber and arguably high sugar intakes suggest that improvements could be made in the quality of carbohydrate choices in athletes with disabilities.

Protein recommendations for AB athletes range from 1.2 to 2.0 g/kg BW depending on type, duration, and intensity of physical activity [3]. Our study found median protein intakes to be 1.6 g/kg BW for males and 1.4 g/kg BW for females, which is similar to others who report 1.7 g/kg BW in male and 1.1 g/kg BW in female athletes with spinal cord injuries [17]. Intakes in this range are likely sufficient, as they fall within the recommended range. Furthermore, it would be expected that these athletes have intakes below the upper limit recommended for AB athletes, as this population uses less active muscle mass and has an overall reduction in energy needs [4]. Further research is required to determine the impact of disabilities on protein needs in athletes.

Dietary fat is important in health and athletics, as it provides the body with energy, aids in the absorption of fat-soluble vitamins, and is a component of cell membranes [3]. Dietary fat intakes as a percent of total calories showed no differences between genders. Fat recommendations for AB athletes suggest intakes should average 20–35% of total calories, with less than 10% coming from saturated fats. Sources of essential fatty acids should be included [3,13]. Total fat intakes fall within the range of 31% of total calories coming from fats. However, saturated fat intakes hover around 10%, suggesting that healthier choices should be encouraged.

### 4.3. Micronutrients

Although no standards are available, it is suggested that athletes may require higher levels of select micronutrients [3], and a diet rich in a variety of foods can help to achieve adequate intakes [1]. Athletes met or exceeded the RDA/AI for the majority of micronutrients, but inadequacies were noted. Our results coincide with others who report deficiencies in calcium, magnesium, folate, and vitamin D in athletes with spinal cord injuries [1,7,17]. It is important to note that these values are from food alone, and the use of supplements may improve intakes.

Calcium and vitamin D have roles in bone-building and muscle strength and function [19,20,21,22]. Females did not meet the recommendation for calcium on average, and neither gender met the recommendations for vitamin D from diet alone. Others have reported insufficient serum vitamin D levels in up to 51% of Canadian and US Paralympic athletes [23] and 27% of Irish Paralympic athletes [22], depending on the season. A study looking at Swiss elite wheelchair athletes reported a high frequency of vitamin D deficiencies all year round; however, deficiencies were greater in the winter and for indoor sports [24]. A vitamin D deficiency can lead to a decrease in neuromuscular function [20]. Furthermore, individuals with spinal cord injuries are at an increased risk of sublesional osteoporosis (SLOP), characterized by excess bone resorption and reduced bone formation [25]. In addition to health concerns, a deficiency may jeopardize sport performance. Compounding the issue is evidence suggesting individuals with physical disabilities are more likely to have insufficient exposure to sunlight. Their skin is more sensitive due to impaired blood flow and thermoregulatory issues in the paralyzed extremities. Consequently, they often wear additional clothing to protect the skin [20]. With low sunlight radiation during winter months in Northern latitudes, it is extremely difficult for athletes with physical disabilities to meet vitamin D requirements [20]. Arguably, greater intakes of vitamin D and an increased reliance on supplementation may be necessary for this population [24,25].

Iron intakes did not meet the RDA, on average, in female athletes. Iron deficiency can promote anemia, which can cause weakness, fatigue, poor concentration, and limit physical performance [26,27]. Female athletes possess a higher risk of iron deficiencies than males due to losses from menses and lower energy intakes [28,29]. In our study, females had significantly lower iron intakes in relation to needs as compared to males, thereby emphasizing the importance of interventions that ensure adequate iron intake in female Paralympic athletes. Inadequacies in dietary iron as well as iron deficiencies have been noted in other female athletic populations [10,29,30], as well as females with spinal cord injuries [7] and female wheelchair basketball players [31], highlighting the potential need for supplementation or nutritional education for female athletes.

Males did not meet the RDA for folate and vitamin A, which have roles in cellular functioning [32] and immunity [33], respectively. Deficiencies in folate and to a lesser degree vitamin A have been reported in male wheelchair basketball players [1]. Neither gender met the AI for potassium, which has a role in muscle cell function [34]. To help alleviate folate, potassium, and vitamin A deficiencies, athletes should eat a varied diet with particular focus on fresh fruits and vegetables. Fortified grain products containing folic acid can also be recommended.

### 4.4. Supplement Use

This study demonstrates that athletes with physical disabilities have a high usage of a wide variety of dietary supplements, as 95% of the participants reported usage over the past three months. To our knowledge, there are few studies investigating supplement use in this population. One study reported 58% of athletes with an impairment consumed dietary supplements within the previous six months [5]. The discrepancy in usage may be due to the inclusion of non-elite athletes in their study, as they note that supplementation was more common in their elite cohort. Comparably, others have found that 87% of elite Canadian AB athletes consumed three or more dietary supplements within a 6 month period [35]. Lower rates have been reported in athletes with spinal cord injuries, with 44% of athletes using supplements at home versus 34% at training camps [7]. The increased dependence on dietary supplements in this most recent study is interesting, and requires further investigation to determine if it is a universal trend or unique to this cohort.

Among the most commonly used supplements were multi-vitamin/multi-minerals, essential fatty acids, and protein powders, which are similar to those reported in other studies [5,6]. Vitamin D was the most common dietary supplement taken, which coincides with the literature reporting deficiencies in athletes with physical impairments [20] and high usage in those with spinal cord injuries [25]. When comparing supplement use to actual micronutrient intakes, athletes met the RDA/AI for most vitamins and minerals. Dietary improvements should bring vitamin and mineral intakes into healthy ranges, negating the need for supplements, with the possible exception of vitamin D. However, not all supplements are taken to increase micronutrient intakes. Supplements such as plant extracts (e.g., Echinacea) are often consumed in the hopes of boosting the immune system [36]. Dietary supplements are also taken to improve performance, for example sport drinks, gels, and gummies are commonly ingested during prolonged exercise to maintain energy levels and blood glucose levels [37].

### 4.5. Sources of Information

It is pivotal to know where athletes seek information on DS in order to develop and implement educational strategies for those involved in the training and education of athletes [38]. Although our findings vary from previous studies [39], a recent study reported similar findings in that athletes with physical impairments receive DS information mostly from dieticians [5]. Studies in AB athletes found family/friends and coaches are common sources [9,40,41]. Coaches were reported as the second-most *preferred* source of nutrition information in the current study. This may be concerning, as athletes may receive false or inaccurate information that is unsupported by research [42]. To counter this risk, coaches should be provided with educational training such as nutritional seminars to enhance their personal nutrition knowledge.

## 5. Conclusions

The information gained from this study is highly relevant to athletes with physical disabilities and their support personnel. High-performance Canadian athletes with physical disabilities could optimize their dietary intakes and supplement use to achieve world-class performance. Future studies should evaluate the impact of the level of impairment on energy and macronutrient intakes to work towards recommendations. In order to adequately support these athletes, educational programs and counseling from sport nutrition professionals are urgently needed.

## Figures and Tables

**Table 1:** Descriptive characteristics.

Descriptive Characteristics	All	Males	Females
Participants	40	18 (45%)	22 (55%)
Age, years	26 (20.5–33.5)	23 (20–31)	27 (23–35)
Weight, kg	n/a	71.7 (10.5)	62.9 (12.7)
Height, m	n/a	1.79 (1.75–1.85)	1.57 (1.55–1.68)
BMI, kg/m^2^	23.5 (3.0)	23.0 (2.8)	24.0 (3.2)
Level of Competition			
Provincial	2 (5.1%)	1 (5.6%)	1 (4.8%)
National	3 (7.7%)	3 (16.7%)	0 (0.0%)
International	34 (87.2%)	14 (77.8%)	20 (95.2%)
Sport Event			
Wheelchair Basketball	27 (67.5%)	11 (61.1%)	16 (72.7%)
Sit Skiing	1 (2.5%)	0 (0.0%)	1 (4.5%)
Para Bobsleigh	1 (2.5%)	1 (5.6%)	0 (0.0%)
Sit Volleyball	2 (5.0%)	0 (0.0%)	2 (9.1%)
Wheelchair Curling	2 (5.0%)	1 (5.6%)	1 (4.5%)
Para Cycling	3 (7.5%)	1 (5.6%)	2 (9.1%)
Para Soccer	1 (2.5%)	1 (5.6%)	0 (0.0%)
Goalball	2 (5.0%)	2 (11.1%)	0 (0.0%)
Para Rugby	1 (2.5%)	1 (5.6%)	0 (0.0%)
Response Rate			
Food Records	36 (90%)	18 (100%)	18 (81.8%)
Dietary Supplements	40 (100%)	18 (100%)	22 (100%)

Descriptive characteristics are provided for all participants. Age and height are median (interquartile range) and weight and body mass index (BMI) are mean (standard deviation). Response rate refers to the percent of participants who completed the food records and dietary supplement portions of the questionnaire, respectively. N/a, not applicable.

**Table 2:** Reported energy and macronutrient intakes.

Nutrient	Total (*n* = 36)	Male (*n* = 18)	Female (*n* = 18)	*p*
Energy, kcal/day	n/a	2092 (1695–2690)	1602 (1439–2059)	**0.013**
Carbohydrates, g/day *	n/a	252.6 (84.9)	209.4 (50.7)	n/a
Carbohydrates, %kcal *	48.0 (8.1)	45.5 (7.2)	50.5 (8.3)	0.064
Carbohydrates, g/kg BW *	3.5 (1.1)	3.5 (1.2)	3.5 (1.0)	0.881
Fiber, g	n/a	22.0 (18–30)	21.0 (16–22)	n/a
Sugar, g *	n/a	99.9 (38.2)	89.6 (23.6)	n/a
Sugar, %kcal *	19.9 (5.8)	18.0 (5.5)	21.8 (5.7)	**0.049**
Protein, g/day	n/a	121.0 (94.4–144.4)	81.7 (59.4–97.4)	n/a
Protein, %kcal *	22.0 (5.4)	24.1 (5.1)	20.0 (5.1)	**0.019**
Protein, g/kg BW	1.5 (1.3–1.7)	1.6 (1.4–2.2)	1.4 (1.1–1.6)	**0.017**
Fat, g/day *	n/a	75.7 (26.8)	57.4 (19.2)	n/a
Fat, %kcal *	30.7 (6.2)	31.0 (6.7)	30.5 (5.9)	0.813
Fat, g/kg BW	1.1 (0.6–1.3)	1.2 (0.6–1.4)	0.9 (0.7–1.2)	0.293
Saturated Fat, g *	n/a	25.5 (9.7)	18.1 (7.4)	n/a
Saturated Fat, %kcal *	10.0 (3.1)	10.5 (3.0)	9.6 (3.1)	0.390
MUFAs, g *	n/a	24.4 (9.1)	20.7 (6.7)	n/a
PUFAs, g	n/a	10.5 (9.0–15.9)	10.4 (7.7–16.7)	n/a
Omega-3, g	n/a	1.1 (0.8–1.3)	1.1 (0.8–1.6)	n/a
Omega-6, g	n/a	2.0 (1.1–3.9)	1.4 (0.6–2.4)	n/a
Trans Fat, g	0.5 (0.2–0.9)	0.5 (0.3–1.5)	0.4 (0.2–0.9)	0.214
Cholesterol, g *	363.1 (193.1)	417.7 (204.4)	308.5 (169.3)	0.090

Intakes are presented as mean (standard deviation) or median (interquartile range). BW, body weight; MUFAs, monounsaturated fatty acids; PUFA, polyunsaturated fatty acids; n/a, not applicable. Percent carbohydrate, sugar, protein, fat and saturated fat are calculated values based on grams of intake. Significant differences between genders in %kcal, g/kg BW, trans fats, and cholesterol were determined by a Mann–Whitney U test. Variables with * are normally distributed, thus an independent *t*-test was performed. *p* < 0.05 was considered significant. Significant differences are bolded.

**Table 3:** Micronutrient intakes from food sources.

Nutrient	Total (*n* = 36)	Male (*n* = 18)	Females (*n* = 18)	*p*
Thiamin, %RDA	102.4 (90.4–153.8)	103.7 (92.2–153.9)	101.6 (88.8–143.0)	0.938
Riboflavin, %RDA	194.8 (144.0–233.9)	231.4 (143.8–258.5)	183.9 (144.3–222.7)	0.339
Niacin, %RDA *	165.6 (68.5)	185.3 (69.0)	146.0 (64.0)	0.085
Vitamin B6, %RDA	170.6 (134.2–254.4)	187.7 (157.1–281.2)	153.4 (118.4–200.9)	0.104
Pantothenic acid, %AI	79.2 (50.0–109.9)	80.8 (49.5–123.5)	72.2 (50.5–98.3)	0.323
Folate, %RDA	105.0 (76.3–126.4)	92.9 (73.4–116.1)	117.2 (83.8–130.8)	0.252
Vitamin B12, %RDA	208.1 (170.6–286.7)	240.1 (187.4–341.4)	189.5 (163.6–224.3)	0.055
Vitamin C, %RDA	123.7 (46.1–176.2)	123.7 (49.9–181.6)	123.7 (42.8–170.8)	0.864
Vitamin D, %RDA	29.4 (11.7–40.8)	35.5 (11.8–42.9)	19.0 (11.3–33.3)	0.265
Vitamin A, %RDA *	103.1 (40.0)	94.7 (46.4)	111.5 (31.4)	0.214
Vitamin E, %RDA	59.4 (49.0–94.0)	59.4 (47.5–98.7)	58.3 (50.5–86.0)	0.628
Calcium, %RDA *	92.8 (35.7)	105.5 (39.2)	80.2 (27.2)	**0.031**
Iron, %RDA	123.0 (70.6–172.5)	172.5 (135.5–280.0)	70.6 (55.6–99.4)	**<0.001**
Magnesium, %RDA	81.9 (63.6–115.5)	80.7 (58.2–116.3)	89.4 (65.7–114.6)	0.628
Selenium, %RDA *	222.2 (80.7)	254.0 (86.3)	190.5 (62.0)	**0.016**
Zinc, %RDA	113.5 (88.4–162.9)	104.6 (88.1–152.7)	128.8 (93.1–177.5)	0.628
Potassium, %AI	59.4 (48.7–74.5)	66.2 (52.3–87.6)	57.3 (45.9–64.3)	0.097
Sodium, %AI	118.8 (86.9–169.8)	146.2 (102.1–196.4)	104.3 (82.3–130.7)	**0.047**
Sodium, %UL	116.6 (83.2–149.4)	135.8 (84.9–191.4)	104.3 (82.3–130.4)	0.097

Intakes are mean (standard deviation) or median (interquartile range). Percent Recommended Dietary Allowance (RDA), Adequate Intake (AI), or Tolerable Upper Intake Level (UL) for each athlete was calculated by taking their intake and dividing it by the established RDA, AI, or UL for that age and gender [13] and multiplying by 100. Differences between genders were determined by a Mann–Whitney U test for data that was not normally distributed or an independent *t*-test for normally distributed data. Variables with * are normally distributed. *p* < 0.05 was considered significant. Significant differences are bolded.

## Supplementary Materials

The following are available online at www.mdpi.com/2072-6643/9/11/1266/s1. Supplementary File S1: Dietary Supplement Questionnaire, Supplementary File S2: Three Day Food Records, Supplementary File S3: Nutrient Intakes, Supplementary File S4: Dietary Supplement Usage, Supplementary File S5: Dietary Supplement Usage Codes, Supplementary File S6: Dietary Supplements and Ergogenic Aids.
