# Supplementary material for: Evaluation of Dietary Intakes and Supplement Use in Paralympic Athletes
Source: Nutrients. 2017 Nov 21;9(11):1266. doi: 10.3390/nu9111266 (PMC5707738; doi:10.3390/nu9111266)
Supplement: Supplementary file 1 [file nutrients-09-01266-s001.zip › S5 Dietary Supplement Usage Codes.pdf]

Variable Values

Value		Label
Gender	0	Male
	1	Female
SportClass	0	endurance
	1	appearance
	2	intermittent
	3	strength
TrainingPhase	0	competition
	1	offseasontrain
	2	offseasonnotrain
LevelComp	0	Provincial
	1	National
	2	International
TrainingHrs	0	0-5
	1	6-10
	2	11-15
	3	15+
UseSuppl	0	No
	1	Yes
ReasonMedical	0	no
	1	yes
ReasonDiet	0	no
	1	yes
ReasonHealthy	0	no
	1	yes
ReasonMuscle	0	no
	1	yes
ReasonEndurance	0	no
	1	yes
ReasonOthers	0	no
	1	yes
ReasonConvenient	0	no
	1	yes
ReasonAllergy	0	no
	1	yes

Variable Values

Value		Label
ReasonEnergy	0	no
	1	yes
ReasonRecovery	0	no
	1	yes
ReasonImmune	0	no
	1	yes
ReasonPerformance	0	no
	1	yes
ReasonTold	0	no
	1	yes
ReasonTaste	0	no
	1	yes
ReasonWeight	0	no
	1	yes
ReasonDietRestrict	0	no
	1	yes
InfoInternet	0	no
	1	yes
InfoPharam	0	no
	1	yes
InfoNaturo	0	no
	1	yes
InfoMD	0	no
	1	yes
InfoAT	0	no
	1	yes
InfoFamily	0	no
	1	yes
InfoTV	0	no
	1	yes
InfoPhysio	0	no
	1	yes
InfoHealthStore	0	no
	1	yes

Variable Values

Value		Label
InfoPrint	0	no
	1	yes
InfoCoach	0	no
	1	yes
InfoDietitan	0	no
	1	yes
InfoLabel	0	no
	1	yes
InfoWorkshop	0	no
	1	yes
InfoTeammates	0	no
	1	yes
InfoNA	0	no
	1	yes
Presentations	0	no
	1	yes
Print	0	no
	1	yes
Consult	0	no
	1	yes
Internet	0	no
	1	yes
Family	0	no
	1	yes
Coach	0	no
	1	yes
HealthStore	0	no
	1	yes
Doctor	0	no
	1	yes
DietRating	0	NotHealthy
	1	Average
	2	VeryHealthy

Variable Values

Value		Label
MVMMCoded	0	Daily
	5	Occasionally
	6	Never
BVitCoded	0	Daily
	5	Occasionally
	6	Never
VitCCoded	0	Daily
	5	Occasionally
	6	Never
VitECoded	0	Daily
	5	Occasionally
	6	Never
VitDCoded	0	Daily
	5	Occasionally
	6	Never
IronCoded	0	Daily
	5	Occasionally
	6	Never
CalciumCoded	0	Daily
	5	Occasionally
	6	Never
MagnesiumCoded	0	Daily
	5	Occasionally
	6	Never
VitWaterCoded	0	Daily
	5	Occasionally
	6	Never
ProteinCoded	0	Daily
	5	Occasionally
	6	Never
SportBarCoded	0	Daily
	5	Occasionally
	6	Never

Variable Values

Value		Label
BCAACoded	0	Daily
	5	Occasionally
	6	Never
GlucosamineCoded	0	Daily
	5	Occasionally
	6	Never
BetACoded	0	Daily
	5	Occasionally
	6	Never
GlutamineCoded	0	Daily
	5	Occasionally
	6	Never
FattyAcidCoded	0	Daily
	5	Occasionally
	6	Never
SportDrinkCoded	0	Daily
	5	Occasionally
	6	Never
EnergyDrinkCoded	0	Daily
	5	Ocassionally
	6	Never
CaffeineCoded	0	Daily
	5	Occasionally
	6	Never
CreatineCoded	0	Daily
	5	Occasionally
	6	Never
RecoveryDrinkCoded	0	Daily
	5	Occasionally
	6	Never
PlantExtractCoded	0	Daily
	5	Occasionally
	6	Never

Variable Values

Value		Label
ProbioticCoded	0	Daily
	5	Occasionally
	6	Never
GelGummiesCoded	0	Daily
	5	Occasionally
	6	Never
