# Supplementary material for: Evaluation of Dietary Intakes and Supplement Use in Paralympic Athletes
Source: Nutrients. 2017 Nov 21;9(11):1266. doi: 10.3390/nu9111266 (PMC5707738; doi:10.3390/nu9111266)
Supplement: Supplementary file 1 [file nutrients-09-01266-s001.zip › S6 Table 4. Dietary supplements and ergogenic aids.docx]

**Table 4.** Dietary supplements and ergogenic aids.

| **Supplement %athletes** | **Total (*n* = 40)** | | | **Males (*n* = 18)** | | | **Females (*n* = 22)** | | | |
| --- | --- | --- | --- | --- | --- | --- | --- | --- | --- | --- |
|  | **R** | **O** | **N** | **R** | **O** | **N** | **R** | **O** | **N** | ***p*** |
| MVMM | 17.5 | 27.5 | 55.0 | 22.2 | 27.8 | 50.0 | 13.6 | 27.3 | 59.1 | 0.753 |
| B Vitamins | 15.0 | 12.5 | 72.5 | 22.2 | 5.6 | 72.2 | 9.1 | 18.2 | 72.7 | 0.301 |
| Vitamin C | 5.0 | 17.5 | 77.5 | 5.6 | 16.7 | 77.8 | 4.5 | 18.2 | 77.3 | 0.983 |
| Vitamin E | 0 | 7.5 | 92.5 | 0 | 5.6 | 94.4 | 0 | 9.1 | 90.9 | 0.673 |
| Vitamin D | 32.5 | 20.0 | 47.5 | 22.2 | 22.2 | 55.6 | 40.9 | 18.2 | 40.9 | 0.451 |
| Vitamin Fortified Water | 0 | 45.0 | 55.0 | 0 | 38.9 | 61.1 | 0 | 50.0 | 50.0 | 0.482 |
| Iron | 10.0 | 17.5 | 72.5 | 5.6 | 5.6 | 88.9 | 13.6 | 27.3 | 59.1 | 0.104 |
| Calcium | 10.0 | 12.5 | 77.5 | 5.6 | 11.1 | 83.3 | 13.6 | 13.6 | 72.7 | 0.657 |
| Magnesium | 0 | 10.0 | 90.0 | 0 | 5.6 | 94.4 | 0 | 13.6 | 86.4 | 0.397 |
| Protein Powder | 30.0 | 32.5 | 37.5 | 38.9 | 27.8 | 33.3 | 22.7 | 36.4 | 40.9 | 0.538 |
| Beta Alanine | 0 | 2.6 | 97.4 | 0 | 6.3 | 93.8 | 0 | 0 | 100.0 | 0.235 |
| BCAA | 0 | 12.5 | 87.5 | 0 | 27.8 | 72.2 | 0 | 0 | 100.0 | **0**.**008** |
| Glutamine | 0 | 8.1 | 91.9 | 0 | 13.3 | 86.7 | 0 | 4.5 | 95.5 | 0.336 |
| Glucosamine | 2.6 | 2.6 | 94.9 | 0 | 5.9 | 94.1 | 4.5 | 0 | 95.5 | 0.355 |
| Fatty Acids | 17.5 | 22.5 | 60.0 | 16.7 | 22.2 | 61.1 | 18.2 | 22.7 | 59.1 | 0.990 |
| Sport Drink | 20.0 | 60.0 | 20.0 | 33.3 | 50.0 | 16.7 | 9.1 | 68.2 | 22.7 | 0.162 |
| Recovery Drink | 5.0 | 20.0 | 75.0 | 5.6 | 22.2 | 72.2 | 4.5 | 18.2 | 77.3 | 0.935 |
| Energy Drink | 7.5 | 37.5 | 55.0 | 11.1 | 44.4 | 44.4 | 4.5 | 31.8 | 63.6 | 0.438 |
| Sport Bar | 25.0 | 47.5 | 27.5 | 38.9 | 44.4 | 16.7 | 13.6 | 50.0 | 36.4 | 0.136 |
| Creatine | 7.5 | 17.5 | 75.0 | 16.7 | 22.2 | 61.1 | 0 | 13.6 | 86.4 | 0.085 |
| Caffeine | 5.0 | 2.5 | 92.5 | 0 | 5.6 | 94.4 | 9.1 | 0 | 90.9 | 0.238 |
| Gel/Gummy | 2.5 | 25.0 | 72.5 | 0 | 22.2 | 77.8 | 4.5 | 27.3 | 68.2 | 0.593 |
| Plant Extracts | 5.0 | 42.5 | 52.5 | 0 | 38.9 | 61.1 | 9.1 | 45.5 | 45.5 | 0.333 |
| Probiotics | 5.0 | 20.0 | 75.0 | 5.6 | 16.7 | 77.8 | 4.5 | 22.7 | 72.7 | 0.889 |

Dietary supplement use is presented as percent of athletes who completed the dietary supplement portion of the questionnaire (n = 40). MVMM, multivitamin-multimineral; BCAA, branched chain amino acids; R, regularly; O, occasionally; and N, never. Significant differences in the percent of athletes in the groups consuming the supplement were determined by a Pearson’s Chi-squared test. P<0.05 was considered significant.
