# Supplementary material for: Evaluation of Dietary Intakes and Supplement Use in Paralympic Athletes
Source: Nutrients. 2017 Nov 21;9(11):1266. doi: 10.3390/nu9111266 (PMC5707738; doi:10.3390/nu9111266)
Supplement: Supplementary file 1 [file nutrients-09-01266-s001.zip › S1 Dietary Supplement Questionnaire.pdf]

Dietary Supplement Questionnaire

Please ensure you sign the consent form prior to completing this questionnaire.

Athlete Name: ___________________________

Age:____ Date of Birth:____________ Gender: M / F Weight: _________ Height:____________

Competitive Sport: ____________________

What training phase/season is this for you?

☐ Competition ☐Off-season training ☐Off-season not training

Please indicate the top level of competition that you have competed in:

☐ Provincial (the province you live in)
☐ National (other provinces within Canada)
☐ International (Countries other than Canada)

On average, how many hours do you train (exercise) each week?

☐ 0– 5 Hours/wk ☐ 6 – 10 Hours/wk ☐ 11-15 Hours/wk ☐ > 15 Hours/wk

Dietary Supplement Use

Do you take any dietary supplements? (e.g. sport drinks like Gatorade, multivitamin, multimineral, vitamins/minerals, protein supplements, sport bars, energy drinks, herbal products, fish oil etc)

☐ Yes ☐ No

How often have you taken the following in the last 3 months? Your choices are: "Regularly" (at least twice per week), "Specific Times" (during competition or when sick), I've Tried it (have taken it before but don't use it regularly), "Never" (you have not tried it ever), or "Unfamiliar" (You don't know what this is) in the "How Often" column. If you do take a supplement please write the exact one in the "Supplement Name" column if you know it.

**Dietary Supplement (Examples)**	**How Often**	**Brand Name and Dose**
Multivitamin & mineral Pill	Regularly Specific Times Tried Never Unfamiliar	
Pill with just B Vitamins (Vitamin B12, Folic Acid, Vitamin B complex, Vitamin B6 Stress Tabs)	Regularly Specific Times Tried Never Unfamiliar	
Pill with just Vitamin C	Regularly Specific Times Tried Never Unfamiliar	
Pill with just Vitamin E	Regularly Specific Times Tried Never Unfamiliar	
Pill with just Vitamin D	Regularly Specific Times Tried Never Unfamiliar	
Other vitamin pills, please list: ____________________	Regularly Specific Times Tried Never Unfamiliar	
Pill with just Iron	Regularly Specific Times Tried Never Unfamiliar	
Pill with just Calcium	Regularly Specific Times Tried Never Unfamiliar	

Pill with just Magnesium	Regularly Specific Times Tried Never Unfamiliar
Other minerals, please list:__________	Regularly Specific Times Tried Never Unfamiliar
Vitaminized Water (Vitamin Water, Aquafina Plus)	Regularly Specific Times Tried Never Unfamiliar
Protein powder (whey/soy/hemp/rice)	Regularly Specific Times Tried Never Unfamiliar
Protein or Sport Bars (Clif Bar, Power bar, Luna bar, Vector bar etc.)	Regularly Specific Times Tried Never Unfamiliar
Branched chain amino acids (BCCA)	Regularly Specific Times Tried Never Unfamiliar
Glucosamine	Regularly Specific Times Tried Never Unfamiliar
Beta-alanine	Regularly Specific Times Tried Never Unfamiliar
Glutamine	Regularly Specific Times Tried Never Unfamiliar
Fatty-acid preparations (Flax seed oil, omega 3 oil or pill, fish oil or pill)	Regularly Specific Times Tried Never Unfamiliar
Sport or electrolyte drinks/supplement (Gatorade, Powerade, Refresh, Nuun, G2, Salt tablets)	Regularly Specific Times Tried Never Unfamiliar
Energy drinks (Red Bull, Rockstar, Monster)	Regularly Specific Times Tried Never Unfamiliar
Caffeine Pills does NOT include coffee (No Doz)	Regularly Specific Times Tried Never Unfamiliar
Creatine (alone or in combination)	Regularly Specific Times Tried Never Unfamiliar
Recovery Drinks (PureSport Recovery, CytoSport Protein Pure, Boost, Ensure, Breakfast Anytime)	Regularly Specific Times Tried Never Unfamiliar
Plant extracts (Echinacea, Cold FX, ginseng, garlic, oil of oregano, beet root extract, rose hip, turmeric)	Regularly Specific Times Tried Never Unfamiliar

Probiotic pills NOT yogurt	Regularly Specific Times Tried Never Unfamiliar
Sport Gels (Powergel, Clif shots, Carb- BOOM).	Regularly Specific Times Tried Never Unfamiliar
Gummy/Bean (Sharkie, Clif Block, Sport Beans)	Regularly Specific Times Tried Never Unfamiliar
Other (Any vitamin, mineral, herb, botanical, amino acid, dietary substance, concentrate, metabolite, constituent or extract not listed above.) Please list:__________________	Regularly Specific Times Tried Never Unfamiliar

What are your reason(s) for taking dietary supplements? Please check all that apply.

a)☐ Medical (your doctor told you to)
b)☐ To improve your diet (food you eat everyday)
c)☐ Stay healthy
d)☐ Increase or maintain muscle mass, strength and/or power
e)☐ Increase endurance (how long you can exercise)
f)☐ Because others (friends, family, teammates) do
g) ☐ Convenient when hungry or thirsty
h) ☐ Food allergy/sensitivity/intolerance
i)☐ Increase energy (so you don't feel tired)
j)☐ Improve exercise recovery (after exercise)
k) ☐ Enhance immune system (so you don't get sick)
l) ☐ Enhance overall athletic performance
m) ☐ Someone told you to (coach, parent, friend)
n) ☐ Enjoy the taste
o) ☐ Weight loss or weight gain
p) ☐ Special dietary needs (i.e., examples?)

Where do you get information about dietary supplements? Please check all that apply.

a)☐ Internet (Websites, Facebook)
b)☐ Pharmacist
c)☐ Naturopath/Chiropractor
d)☐ Medical Physician (Doctor)
e)☐ Athletic Trainer
f)☐ Family
g)☐Television
h)☐ Physio/Massage Therapist
i)☐ Health Food Store
j)☐ Print Media (magazines, books)
k)☐ Coach
l)☐ Dietitian/ Nutritionist
m)☐ Product Labels
n)☐ Workshops/Classes
o)☐ Teammates/Friends
p)☐ Not Applicable (I do not take dietary supplements)

Which way do you prefer to receive information about dietary supplements? Please rank UP TO 3 responses with "1" as your "FIRST CHOICE":

☐ Presentations
☐ Print media (pamphlets, books, magazines)
☐ Individual Nutrition Consultation (dietitian)
☐ Internet or e-mail
☐ Family/friends
☐ Coach or athletic trainer
☐ Health Food Store/Pharmacy
☐ Doctor/chiropractor/physiotherapist etc.

How would you rate your diet (how healthy you eat everyday)?

Not Very Healthy ☐   Pretty Healthy (average) ☐   Very Healthy ☐
