# Supplementary material for: Evaluation of Dietary Intakes and Supplement Use in Paralympic Athletes
Source: Nutrients. 2017 Nov 21;9(11):1266. doi: 10.3390/nu9111266 (PMC5707738; doi:10.3390/nu9111266)
Supplement: Supplementary file 1 [file nutrients-09-01266-s001.zip › S2 Three Day Food Records.pdf]

THREE-DAY DIETARY INTAKE RECORD

Name:_________________________________
(full name)

Date of Birth: _____ ______ ______
(Day) (Month) (Year)

Height: ___________ Weight: ______________

Vegetarian (please circle): Yes No

Competitive Sport: _______________

What training phase/season is this for you?

- Competition
- Off-season training
- Off-season not training

e-mail address: _________________________
(required for us to send you your complete diet analysis)

Please ensure you sign the consent form prior to completing this questionnaire. We require a signed copy of the consent form in order to analyse your results and provide you with your personalized diet report. Thank you!

Instructions

The purpose of this research project is to evaluate dietary intakes in Paralympic athletes. It is important to record ALL foods and beverages – whether it is a full course meal at home or a quick sport drink at training. Before you start recording your intake, please read the following instructions and review the instructional videos on our website http://blogs.mtroyal.ca/athletes/. It is a good idea to carry your Dietary Intake Record book with you and record your entries as soon after eating as possible. Foods and beverages consumed away from home – at a friend's house, at training, at a restaurant- are just as important as those eaten at home.

Please include the following information for each of ***Day 1, Day 2 and Day 3*** of your Food Record:

1. **DAY/DATE INFORMATION:** Fill in the section on the top of each page to indicate the date and day of the week.
2. **MEAL/TIME** column: Indicate the meal and time the food was eaten. Snacks can be entered wherever appropriate on the sheet. Water can be recorded at the end as total "throughout the day".
3. **FOOD AND BEVERAGE ITEMS** column: Enter all foods and beverages consumed at the meal or snack time. Please record the specific type of food (for example: *WHOLE WHEAT* bread, *FROSTED FLAKES* cereal). In the same column, record all toppings or items added at the time of eating (for example: sugar, syrup, jam, butter, mayonnaise, gravy, milk, salt, etc.). For combination foods, please include detailed information on each item. For example: If you had a tuna sandwich, you would list the following foods and include detailed information for each of them: white bread, mayonnaise, celery, solid white tuna packed in water, salt.
4. **DESCRIPTION OF ITEM** column: Every food requires a description, **DO NOT** leave this column blank. For every food or beverage item listed, include the following (if applicable):
   - **Brand**: *MIRACLE WHIP* mayonnaise, *PIZZA HUT DEEP DISH* pizza, *OREO* cookie
   - **Type of flavour**: *BLUEBERRY* muffins, *STRAWBERRY* yogurt
   - **Methods of cooking**: *FRIED, BAKED, BBQ'D, HOMEMADE*
   - **Other relevant information on the food label:** *LOW FAT* ranch salad dressing, *28% M.F. (MILK FAT*) cheddar cheese, *LEAN* Ground Beef
5. **AMOUNT** column: For every item consumed, enter the amount eaten using specific units of measure that are convenient for you. For example: enter the word "cup", "grams", "piece", "ounce", "number", "teaspoon", or "tablespoon". Enter a unit of measure not only for the menu item, but for toppings or items added as well. Each entry must have its own unit of measure. Use measuring cups and spoons whenever possible during the 3-day period to record the most accurate estimation of the amount of each food you consume.

   To estimate portion sizes, use the guidelines below:

This amount of food: ……………	is about the same size as:
2½ oz (75g) of meat	a hockey puck
1½ oz (50 g) of cheese	2 white erasers
1 cup (250 mL)	a baseball or fist
½ cup (125 mL)	a hockey puck
1 medium piece of fruit	a tennis ball
2 Tbsp (30 mL)	1 golf ball
¼ cup (60 mL)	2 golf balls
1 tsp (5 mL) – use for butter, margarine, mayonnaise	a thumb tip or one die

Daily check: in the evening, after you have recorded everything for the day, go back over your entries to make sure you have included as much detail as possible for each item.

Note: All foods and beverages you consume every day are important and your Dietary Intake Record should be as accurate as possible. It should also reflect the way you usually eat.
Important! Please do not change your normal eating habits for the 3 days you are recording your food intake. Your honesty is crucial to the quality of diet analysis report you will receive through participating in this research project.

Thank You!!

Food Record Sheets

SAMPLE DAY

Day 3

Date: January 20, 2014

Day of the week (circle): M T W (Th) F Sa Su

Meal/Time (Breakfast, lunch, dinner, 8am, 11am)	***Food/Beverage*** Enter all foods and beverages consumed. For combination foods, please include detailed information on each item.	***Description*** Include a detailed description of each item including: brand name, flavor, cooking method.	***Amount*** Enter amount and unit of measure for example: cup, GRAMS, OUNCE, piece, teaspoon (tsp), tablespoon (tbsp).
Breakfast	Cereal	Life multigrain (1/4c dry 39g)	1 cup
9 am	Milk	1% Dairyland	175ml 3/4 cup
Snack	Oatmeal cookies	Dad's Oatmeal cookies	2
11 am			
Lunch	PB & J sandwich:		
12:45pm	whole wheat bread	Dempsters 100%	1 slice
	peanut butter	President's Choice (PC) organics	2 tbsp
	jam strawberry	Smuckers	2 tbsp
	Orange juice	PC from concentrate	1 cup
	Apple	Spartan	1
Snack	Tim Horton's coffee	Black	250 mL
3pm	cream	half & half	1 tbsp
	sugar		1 tsp
Dinner	Spaghetti & meat sauce (restaurant) Chianti's		
6PM	pasta	whole wheat	1.5 cup
	meat sauce	restaurant type (beef)	1 cup
	parmesan cheese	" "	1 tbsp
	Ceasar salad		
	lettuce	Romaine	1.5 cup
	croutons		2 tbsp
	bacon bits	restaurant type	2 tbsp
	dressing (cesar)	" "	~1.5 tbsp

Food Record Sheets

Day 1

Date:___________________________ **Day of the week** (circle): M T W Th F Sa Su

Meal/Time (Breakfast, lunch, dinner, 8^{am}, 11^{am})	***Food/Beverage*** Enter all foods and beverages consumed. For combination foods, please include detailed information on each item.	***Description*** Include a detailed description of each item including: brand name, flavor, cooking method.	***Amount*** Enter amount and unit of measure for example: cup, GRAMS, OUNCE, piece, teaspoon (tsp), tablespoon (tbsp).

Day 1 Continued

Meal/Time<br>(Breakfast, lunch, dinner, 8^{am}, 11^{am})	***Food/Beverage***<br>Enter all foods and beverages consumed. For combination foods, please include detailed information on each item.	***Description***<br>Include a detailed description of each item including: brand name, flavor, cooking method.	***Amount***<br>Enter amount and unit of measure for example: cup, GRAMS, OUNCE, piece, teaspoon (tsp), tablespoon (tbsp).

Day 2

Date:__________________________ **Day of the week** (circle): M T W Th F Sa Su

Meal/Time (Breakfast, lunch, dinner, 8^{am}, 11^{am})	***Food/Beverage*** Enter all foods and beverages consumed. For combination foods, please include detailed information on each item.	***Description*** Include a detailed description of each item including: brand name, flavor, cooking method.	***Amount*** Enter amount and unit of measure for example: cup, GRAMS, OUNCE, piece, teaspoon (tsp), tablespoon (tbsp).

Day 2 Continued

Meal/Time<br>(Breakfast, lunch, dinner, 8am, 11am)	***Food/Beverage***<br>Enter all foods and beverages consumed. For combination foods, please include detailed information on each item.	***Description***<br>Include a detailed description of each item including: brand name, flavor, cooking method.	***Amount***<br>Enter amount and unit of measure for example: cup, GRAMS, OUNCE, piece, teaspoon (tsp), tablespoon (tbsp).

Food Record Sheets

Day 3

Date:__________________________ **Day of the week** (circle): M T W Th F Sa Su

Meal/Time (Breakfast, lunch, dinner, 8^{am}, 11^{am})	***Food/Beverage*** Enter all foods and beverages consumed. For combination foods, please include detailed information on each item.	***Description*** Include a detailed description of each item including: brand name, flavor, cooking method.	***Amount*** Enter amount and unit of measure for example: cup, GRAMS, OUNCE, piece, teaspoon (tsp), tablespoon (tbsp).

Day 3 Continued

Meal/Time<br>(Breakfast, lunch, dinner, 8^{am}, 11^{am})	***Food/Beverage***<br>Enter all foods and beverages consumed. For combination foods, please include detailed information on each item.	***Description***<br>Include a detailed description of each item including: brand name, flavor, cooking method.	***Amount***<br>Enter amount and unit of measure for example: cup, GRAMS, OUNCE, piece, teaspoon (tsp), tablespoon (tbsp).
